# Electric field tuning of spin splitting in topological insulator quantum dots doped with a single magnetic ion

**DOI:** 10.1038/s41598-019-45067-5

**Published:** 2019-06-24

**Authors:** Xiaojing Li, Wen Yang, Liangzhong Lin, Zhenhua Wu

**Affiliations:** 1 College of Physics and Energy, Fujian Normal University, Fuzhou, 350007 China; 2 Beijing Computational Science Research Center, Beijing, 100089 China; 3 Key Laboratory of Microelectronic Devices and Integrated Technology, Institute of Microelectronics, Chinese Academy of Sciences, Beijing, 100029 China; 4 R&D center, Shenzhen Puyi Lighting Technology Ltd, Shenzhen, 518000 China

**Keywords:** Quantum dots, Electronic properties and materials

## Abstract

We investigate theoretically the electron spin states in a disk-shaped topological insulator quantum dot (TIQD) containing a single magnetic *Mn*^2+^ ion. We demonstrate that the energy spectra and the density distributions of the symmetry-protected edge states in a HgTe TIQD can be modulated effectively by a single magnetic impurity *Mn*^2+^. Additionally, when an in-plane external electric field is applied., it not only tunes the spin splittings of edge states via the *s*(*p*)-*d* exchange interaction between the electron (hole) and the magnetic *Mn*^2+^ ion respectively, but also gives rise to the bright-to-dark transitions in the optical transition spectrum. Such spin properties of TIQDs with single *Mn*^2+^ ion as illustrated in this work could offer a new platform for topological electro-optical devices.

## Introduction

The topological insulator (TI) is a novel class of quantum matters with insulating bulk characterized by *Z2* topological invariant, and metallic edges or surfaces protected by time reversal symmetry (TRS)^1–4^. The edge states or surface states of TIs are robust against nonmagnetic impurities or crystal defects and thus are fundamentally different from the bulk states. The two-dimensional (2D) TIs were theoretically proposed and experimentally discovered in HgTe quantum wells (QWs), that exhibit a quantum phase transition as the thickness of QWs (*d*_*QW*_) increases^5^. The band structure of HgTe QWs is inverted when ${d}_{QW} > {d}_{c}$, i.e., the *p*-type ${{\rm{\Gamma }}}_{8}$ band lies above the *s*-type ${{\rm{\Gamma }}}_{6}$ band, where ${d}_{c}=6.3\,nm$ is the critical thickness. Gapless helical edge states in HgTe QWs have been demonstrated experimentally via the conductance plateau when the Fermi energy is tuned inside the bulk gap^6^. Since then, intensive efforts have been devoted realize novel device applications utilizing the TI states^7–15^. Besides HgTe and InAs/GaSb^16^ QWs, conventional semiconductors GaN/InN/GaN and GaAs/Ge/GaAs QWs could also be driven into topological phases utilizing interface polarizations or external field^17–19^.

The robustness of the edge states against external perturbations has been established, except for a strong magnetic field. In TIs doped with magnetic impurities, one can expect to see the interplay between the spin-orbit interactions (SOIs) and *sp-d* exchange interaction, which could lead to interesting phenomena, e.g., twisted RKKY interaction^20^ and quantum anomalous Hall effect^21^. The HgTe QWs doped with *Mn*^2+^ ions, in which magnetic moments induce an effective nonlinear Zeeman effect, cause a non-monotonic bending of the Landau levels^22^. Due to the breaking of time-reversal symmetry and rotation symmetry, edge states will disappear with high concentration doping. However the impacts on topological edge states by only one or very few magnetic dopants have been rarely studied. It is appealing to investigate such topological insulator quantum dots doped with a single magnetic ion. If the TI edge states sustain in the presence of a single magnetic dopant, one can make use of such configuration to realize the tunable TI quantum device instead of applying magnetic field.

Compared with TI QWs, other TI nanostructures such as TI quantum dot (TIQD) doped with magnetic impurities are rarely explored both theoretically and experimentally. Semiconductor quantum dots (QDs) have attracted intensive research interests in the past decades due to their wide applications in electronic devices, e.g., diode lasers and solar cells^23–25^. For various conventional semiconductor QDs, the conduction band lies above the valence band, i.e., normal band alignment. The electron and hole ground states are both located at the central region of the QDs^26–28^, therefore the QDs display interband optical transitions with strong oscillator strengths. By doping magnetic ions into these semiconductor QDs, spin splitting and spin-relevant optical property can be changed significantly due to strong *sp*-*d* exchange interactions between electrons or holes and magnetic ions^29,30^. QDs doped with a single *Mn*^2+^ ion have been realized experimentally^31,32^. The interband transition in such QDs can be observed clearly by photoluminescence (PL) spectra^33–37^. The magnetic ion doped QDs can produce a promising platform to couple the electronic, optical and spintronic applications^38^. On the other hand, it is appealing to systematically address the novel electronic and optical characteristics of a magnetic ions doped TIQDs in the presence of the aforementioned spin polarized topological edge states. For a TIQD without any magnetic dopant, the helical edge states in a 2D TI are quantized along the circumferences of the TIQDs as whispering gallery modes in photon cavity, leading to interesting equally-spacing edge states. In the presence of a perpendicular magnetic field, several studies reported persistent currents in the TIQDs and oscillations in the magnetic moment of Dirac electrons with increasing magnetic fields, ie., the Aharonov-Bohm effect^39–41^. As an extension of previous studies, in this work, we focus on the spin polarized topological edge states that are modulated by the exchange interactions between a single magnetic dopant and the topological edge states as well as an external in-plane electric field. We also propose a practical way to detect such modulations by optical measurement. TIQDs with a single magnetic dopant offer us the possibility of designing the topological electro-optical devices. It also plays a potential platform to observe Quantum Anomalous Hall Effect (QAHE) in QDs, which is beyond the scope of this work.

In the proposed disk-shaped HgTe quantum dot. The edge states show spin angular momentum locking and ringlike density distributions. In the presence of a single *Mn*^2+^ ion, most edge states disappear due to the breading of rotational symmetry, while some edge states with small angular momentum still exist. The energies of edge states can be effectively tuned by the positions of the single *Mn*^2+^ ion and an external in-plane electric fields. It offers us a new way to tune the electronic and optical properties of the TIQD. The edge states of the TIQD with opposite spin orientation and angular momentum in a such TIQDs exhibit different responses to the doped magnetic ion due to the *s(p)-d* exchange interactions between the electron (hole) and *Mn*^2+^ ion respectively. We further find that the spin-splitting of *e* − *h*-pair energy spectra that can be tuned by the electric fields and the position of doped *Mn*^2+^ ion. Finally, our numerical results indicate that transition optical transition rate of edge states in the TIQD varies from bright to dark as the electric field and the position of *Mn*^2+^ ion varies.

## Methods

**Figure 1:**
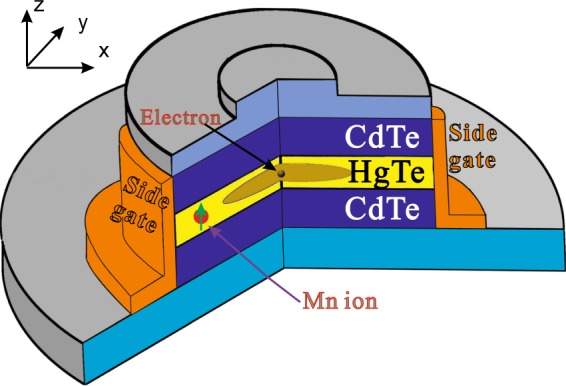
Schematic diagram of a disk-like HgTe topological insulator quantum dot(TIQD) doped with a single *Mn*^2+^ ion.

We consider a HgTe TIQD^39^ doped with a single *Mn*^2+^ ion as shown in Fig. 1. The inverted band structure of HgTe TIQD in the low-energy regime can be described by the Bernevig-Hughes-Zhang(BHZ) model^5^, i.e., a four-band Hamiltonian obtained from the eight-band Kane model with neglecting the light-hole bands as shown below:

1 $${H}_{eff}({k}_{x},{k}_{y})=(\begin{array}{cc}H(k) & 0\\ 0 & {H}^{\ast }(\,-\,k)\end{array})+{H}_{sp-d}+(V(\rho )+{H}_{ele}){I}_{4\times 4},$$

where $H(k)=\varepsilon (k)+{d}_{i}(k){\sigma }_{i}$, *σ*_*i*_ are the Pauli matrices, The elements in the first Hermitian matrix term are given by,

2 $${d}_{1}+i{d}_{2}=A({k}_{x}+i{k}_{y})\equiv A{k}_{+},$$

3 $${d}_{3}=M-B({k}_{x}^{2}+{k}_{y}^{2}),$$

4 $${\varepsilon }_{k}=C-D({k}_{x}^{2}+{k}_{y}^{2}),$$

where $k=({k}_{x},{k}_{y})$ is the in-plane momentum of electrons and *A*, *B*, *C*, and *D* are material and structure-relevant parameters. The relevant material parameters used in our calculation are $A=-\,0.342\,eV\cdot nm$, $B=-\,0.169\,eV\cdot n{m}^{2}$, $C=-\,0.00263\,eV$, $D=0.00514\,eV\cdot n{m}^{2}$, and $M=-\,0.03\,eV$ respectively. Note that the sign of the parameter *M* characterizes the topological insulator phase, which is determined by the thickness of the HgTe TIQD. In this work, we focus on the response of topological edge states to the single magnetic dopant *Mn*^2+^ ion as well as the external electric fields, thus *M* is set to be negative. This four-band Hamiltonian is in the band-edge Bloch basis $|E,1/2\rangle$, $|HH,3/2\rangle$, $|E,-\,1/2\rangle$, $|HH,-\,3/2\rangle$. $|E\rangle$ ($|HH\rangle$) stand for electron-like (hole-like) branch respectively. *H*_*sp*–*d*_ is due to the *sp*–*d* exchange interactions. In eight-band Kane model, we can consider the *sp-d* exchange interaction between electron and magnetic *Mn*^2+^ ion in the HgTe TIQD in the form:

5 $${H}_{sp-d}=-\,\frac{1}{2}J(r-{R}_{M}){S}_{M}\cdot {\sigma }_{e},$$

*σ*_*e*_ is the spin operator of the electrons at the position *r*, ${S}_{M}=|5/2,{(5/2)}_{z}\rangle$ is the spin operator of the *Mn*^2+^ ion at *R*_*M*_ which has six components. $J(r-{R}_{M})$ is the electron-ion exchange integral, *R*_*M*_ is the position of *Mn*^2+^ ion. This term can be written as $\langle S|J(r-R)|S\rangle =\alpha \delta (r-R)$ and $\langle \,j|J(r-R)|j\rangle =\beta \delta (r-R)$ in our basis. *α* (*β*) is the exchange coefficient of the electron in the conduction (valence) band with the magnetic ion in eight-band model. The exchange parameter of the electron in the conduction(valence) band is $\alpha =0.4\,eV$ $(\beta =-\,0.6\,eV)$ in full Kane model^22^. In our four-band model,the hole-like band $|HH,\pm \,3/2\rangle$ is the heavy hole ${{\rm{\Gamma }}}_{7}$, while the electron-like band $|E,\pm \,1/2\rangle$ is a linear combination of electron ${{\rm{\Gamma }}}_{6}$ and light hole ${{\rm{\Gamma }}}_{8}$ in eight-band Kane model. We can write the *s-d* interaction in the same form but with the new basis^42^. Thus the exchange parameters *α* should be represented by $\alpha ^{\prime} ={F}_{1}\alpha +{F}_{2}\beta$, where $\alpha ^{\prime} =0.174\,eV$. *F*_1_ and *F*_2_ is the coupling parameters depending on different well thicknesses and different *Mn*^2+^ doping, which can be calculated from the full Kane model at small *k*^22,43^. The *β* in four band model stays the same as in Kane mo_*d*_el. The *H*_*sp*−*d*_ can be finally rewritten in the matrix form in the new basis vector as:

6 $${H}_{sp-d}=[\begin{array}{cccc}1/2\alpha ^{\prime} {S}_{i} & 0 & 1/2\alpha ^{\prime} {S}_{i}^{-} & 0\\ 0 & 3/2\beta {S}_{i} & 0 & 0\\ 1/2\alpha ^{\prime} {S}_{i}^{+} & 0 & -\,1/2\alpha ^{\prime} {S}_{i} & 0\\ 0 & 0 & 0 & -\,3/2\beta {S}_{i}\end{array}].$$

The confining potential $V(\rho )$ of TIQDs can be defined as a hard-wall potential: $V(\rho )=0$, for $\rho  < R$, and $\infty$ for else where. $R=50\,nm$ is the radius of HgTe TIQD. *H*_*ele*_ is the in-plane electric field $\vec{E}$ applied across the TIQD, then the electrostatic potential can be described as ${H}_{ele}=e\vec{E}\cdot \vec{r}$. *I*_4×4_ is the identity matrice.

The eigenstates and eigenenergies can be obtained numerically by expending the envelop wave function in terms of the Bessel basis, ${{\rm{\Psi }}}_{i}={\sum }_{n,m}\,{C}_{n,m}^{(i)}{\phi }_{n,m}$, in which ${C}_{n,m}^{(i)}$ is the expanding coefficient. For a hard-wall circular disk TIQD, the basic function ${\phi }_{n,m}$ can be expressed as,

7 $${\phi }_{n,m}={N}_{C}\,{J}_{m}({k}_{n}^{m}\rho /R){e}^{im\varphi },$$

in which ${k}_{n}^{m}$ is the *n* the zero point of the first kind of the cylinder Bessel function *J*_*m*_(*x*), ${N}_{C}=1/[\sqrt{\pi }R{J}_{m+1}({k}_{n}^{m})]$, $m=0,\pm \,1,\pm \,2\cdots$ is the quantum number of the angular momentum. After considering this exchange interaction, the new basis vector of the Hamitonian *H* can be written as the direct-product of *H*_0_ basis vector and spin state of *Mn*^2+^ ion as $|e\rangle \otimes |{S}_{M}^{z}\rangle$ and $|hh\rangle \otimes |{S}_{M}^{z}\rangle$.

The interaction Hamiltonian between the Dirac fermion and the photon within the electrical dipole approximation is ${H}_{{int}}=H(\vec{p}+e\vec{{\mathscr{A}}})-H(\vec{p})$, where the vector potential $\vec{{\mathscr{A}}}=({{\mathscr{A}}}_{x}\pm i{{\mathscr{A}}}_{y}){e}^{-i\omega t}$ corresponds to the *σ*± circularly polarized lights. We suppose that the fermi level locates in the middle of the bandgap. $|i\rangle$ denotes the initial states in the lower cones that are hole like bands below the fermi level, $|f\rangle$ denotes the final states in the upper cones that are electron like bands above the fermi level. Then the electron-hole (e-h) pairs are generated via light-matter interaction induced transition from state $|i\rangle$ to state $|f\rangle$. $|i\rangle$ and $|f\rangle$ correspond to the eigenstates of conduction band and valence band ${{\rm{\Psi }}}_{e,h}$ that we have obtained above. The resulting optical transition rate, i.e., *e* − *h* pair generation rate is given by,

8 $${w}_{if}=2\pi \delta ({E}_{f}-{E}_{i}-\hslash \omega )|\langle f|{H}_{{int}}|i\rangle {|}^{2},$$

in which $$\langle f|{H}_{{int}}|i\rangle ={\sum }_{{n}_{1},{m}_{1},{n}_{2},{m}_{2}}\,{C}_{f,{n}_{1},{m}_{1}}^{+}{\phi }_{{n}_{1},{m}_{1}}^{\ast }{H}_{{int}}{C}_{i,{n}_{2},{m}_{2}}{\phi }_{i,{n}_{2},{m}_{2}}$$.

## Discussion

**Figure 2:**
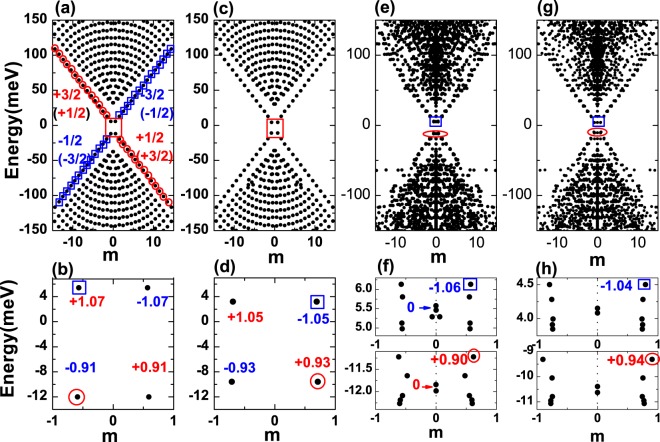
(**a**) The energy spectra of HgTe TIQDs versus the orbital angular momentum $m=\langle {L}_{Z}\rangle$, the edge states with spin up (spin down) are marked by the red circles (blue rectangles). (**b**) The edge states and their average spin $\langle {S}_{z}\rangle$ near the fermi surface in the red square region are enlarged in (**b**). (**c**) The same as (**a**), but with in-plane electric field $E=4\,kV/cm$ along the *x* direction, the edge states in red square region are enlarged in (**d**). (**e**) The same as (**a**), but with a single *Mn*^2+^ magnetic ion located at the edge of the TIQD, the edge states and corresponding average spin $\langle {S}_{z}\rangle$ in red and blue circles are shown in detail in (**f**). (**g**) The energy spectra when the in-plane electric field and a single *Mn*^2+^ magnetic ion located at the edge of the TIQD, the edge states and corresponding average spin $\langle {S}_{z}\rangle$ denoted in red and blue circles are shown in detail in (**h**).

First we illustrate the variations of energy spectra of a HgTe TIQD in the presence of an in-plane electric field and a single magnetic dopant *Mn*^2+^ ion in Fig. 2. The edge states of TIQD show an approximate linear energy-angular-moment dispersion except for a small gap of about 18 *meV* near the Dirac point (see Fig. 2(a)). The spin-up (spin-down) edge states are denoted by the red circles (blue rectangles). Note that the dominant spin elements of edge states in the conduction band are $|\,\pm \,3/2\rangle$ (i.e., the *HH* branch), while the dominant spin components of edge states in valence band are $|\,\pm \,1/2\rangle$ (i.e., the *E* branch), arising from the band inversion of HgTe quantum well. From the edge state near Fermi surface denoted by the red square in Fig. 2(a), we can find the dominant spin component of edge state is $|\,-\,3/2\rangle$ ($|\,+\,\mathrm{3/2}\rangle$) when angular quantum number *m* is positive (negative) respectively. It means that electrons with opposite spin orientations propagate along opposite directions, i.e., the spin-angular-momentum locking. The off-diagonal elements in the first term of Hamiltonian couple the $|E,1/2\rangle$ and the $|HH,3/2\rangle$, (or the $|E,-\,1/2\rangle$ and the $|HH,-\,3/2\rangle$) states respectively. Quantitatively, the average spins of the lowest edge states (average of the four spin states $|E,\pm \,1/2\rangle$, $|HH,\pm \,3/2\rangle$) are calculated and given in 2(b). When we apply an external in-plane electric field along the *x* direction, both the edge states and bulk states are affected significantly as shown in Fig. 2(c). The in-plane electric field changes the energy spectra of TIQD in two different ways: (1) Due to the *Stark* effect, the electric field decreases the bandgap of the TIQD from 18 *meV* to 13 *meV*. (2) The electric field changes the average spin of the edge states by increasing the coupling between the electrons and heavy-hole states (see Fig. 2(d)).

Next we discuss the impacts of the *Mn*^2+^ ion located at the edge of the TIQD ($R=47\,nm$) on the energy spectrum of TIQD as shown in Fig. 2(e). For this dopant position, the *sp-d* interaction between the *Mn*^2+^ ion and the edge states is maximized, giving rise to the largest spin splittings in the edge states. The existence of *Mn*^2+^ ion breaks the time-reversal symmetry and rotation symmetry of the TIQD, and thus the total angular momentum is no longer a good quantum number. However, we still label the different states by their average angular momentum, due to the very weak *sp-d* interaction. We can still observe the edge states clearly when the angular momentum *m* is small. It is a good manifestation of the TI states which are robust against low density magnetic impurities. The edge states at the bottom of conduction band and the top of valence band are shown in detail in Fig. 2(f). The edge states split both in energy and angular momentum. We note that the edge states in the conduction (or valence) bands are split into twelve states corresponding to the coupling states between hole-like spin (or electron-like) and *Mn*^2+^ spin, accounting for the band inversion of HgTe quantum well. Such energy splits arise from the giant *Zeeman* effect, i.e., the diagonal elements of the *s*–*d* and *p*–*d* interactions. As the angular momentum *m* approaching 0, spin states are strongly mixed. The magnitudes of average spins of the edge states is almost 0, which means the probability of holes distributed in $|+1/2\rangle$ spin and $|-1/2\rangle$ spin states in the valence band (or electrons distributed in $|+3/2\rangle$ spin states and $|-3/2\rangle$ spin states in conduction band) are almost the same. The horizontal splitting of energy spectrum comes from the off-diagonal elements of the *s*–*d* interaction, inducing the coupling between the states $|e,Sz\rangle$ and $|e\pm 1,Sz\mp 1\rangle$ via simultaneous spin flip of the the *Mn*^2+^ ion and electrons. The horizontal shifts of the valence band are larger than that in conduction band. Because the main spin component in valence band is ±1/2 which can couple with the *Mn*^2+^ ion through the off-diagonal elements while the electrons in conduction band with main spin ±3/2 can not flip together with the *Mn*^2+^ spin. Depend on the *Mn*^2+^ ion, the maximum spin splitting of edge states in conduction band or valence band is nearly 1 *meV*.

**Figure 3:**
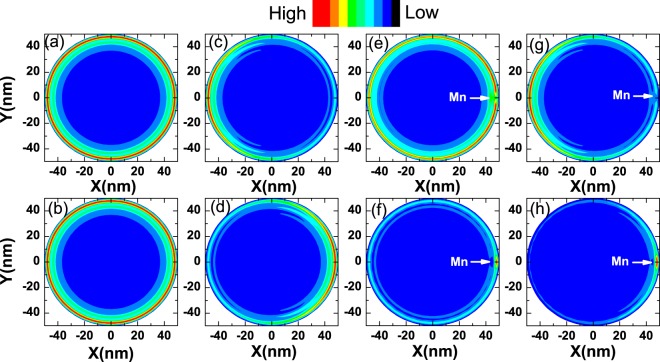
(**a**,**b**) The density distributions of the edge states at the bottom of conduction band and at the top of the valence band marked by the blue and red circles in Fig. 2(b) respectively. (**c**,**d**) Denote the states shown blue and red circles in Fig. 2(d) with an in-plane electric field. (**e**,**f**) Denote the states shown blue and red circles in Fig. 2(f,g) with a *Mn*^2+^ magnetic ion located at the edge. (**g**,**h**) Show the distributions of the edge states with both in-plane electric field and *Mn*^2+^ magnetic ion located at the edge.

To realize the tunable spin splitting, we apply an electric field in the plane (along the *x* direction) of the TIQD with a single *Mn*^2+^ ion dopant. The energy-angular-moment dispersion is shown in Fig. 2(g). We enlarge the plot of spin splitting in Fig. 2(h). We can find the spin splitting of the edge state in valence band increases to 2 *meV*, while the spin slitting for edge state in conduction decrease to 0.5 *meV*. Since the in-plane electric field pushes the wavefunction of the edge states in conduction away from the *Mn*^2+^ ion, and decreases the overlap between the electrons and the magnetic ion. The effect of electric field on the edge states in valence band is opposite. We can tune the spin splitting of the edge states through the external electric field. Simultaneously, the angular momentum shifts of edge states can also be affected by the electric field. These effects can be confirmed by investigating the carrier density distributions as we will discuss in detail about Fig. 3.

The spin splitting changed by the electric field can be understood from the density distributions of carriers in the edge states at the bottom of the conduction band and at the top of the valence band as shown in Fig. 3. In Fig. 3(a,b), we plot the density distributions of edge states of the TIQD denoted by blue rectangle and red circle in Fig. 2(b) (the edge states from conduction and valence band). In the absence of external electric field, it shows ring-like distributions. When an in-plane electric field is applied, it pulls the electrons of edge state at the bottom of conduction band to the left side as shown in Fig. 3(c), while pushes the holes of edge state at the top of valence band to the opposite direction as shown in Fig. 3(d), i.e., classic responses of the TI edge states to the in-plane electric field. When we dope a *Mn*^2+^ ion near the edge of this TIQD, the density distributions of edge state denoted by the blue rectangle and red circle in Fig. 2(f) are shown in Fig. 3(e,f). The carrier distribution of edge state of the bottom conduction band still exhibits ring-like behavior but with centroid away from the *Mn*^2+^ ion. The spin orientations are different between electrons and the *Mn*^+2^ ion. Due to the ferromagnetic *p-d* exchange interaction, there is a repel interaction between the electron and the *Mn*^+2^ ion. In contrast, the carrier density distribution of edge state of the top valence band shows opposite trend, i.e., holes are located close to the *Mn*^2+^ ion as shown in Fig. 3(f). It arises from the same spin orientations between carrier in valence band and the *Mn*^+2^ ion. The antiferromagnetic *s-d* exchange interaction results in an attractive force between the carrier in the valence band and the *Mn*^+2^ ion. The *Mn*^2+^ ion behaves like a scattering center for the edge states. When we consider an in-plane electric field applied across such a *Mn*^2+^ ion doped TIQD, we can see the electric field pulls the edge state in conduction band to the left side away from the *Mn*^2+^ ion, while it pushes the carriers of edge state in valence band to the right side close to the *Mn*^2+^ ion as shown in Fig. 3(g,h), respectively. According to the carrier distributions in the presence of an in-plane electric field, the aforementioned spin splittings of edge states due to the *sp-d* interaction in the Hamiltonian between electron/hole and the *Mn*^2+^ ion depends on the overlap between the wavefunctions of electron/hole and the *Mn*^2+^ ion. So the in-plane electric field will increase the overlap of wavefunction and strength of *p*–*d* interaction, resulting in the larger spin splitting in valence bands as shown in lower panel of Fig. 2(h). On the contrary, it decreases the *s*–*d* interaction and the spin splittings in conduction bands as shown in the upper panel of Fig. 2(h).

**Figure 4:**
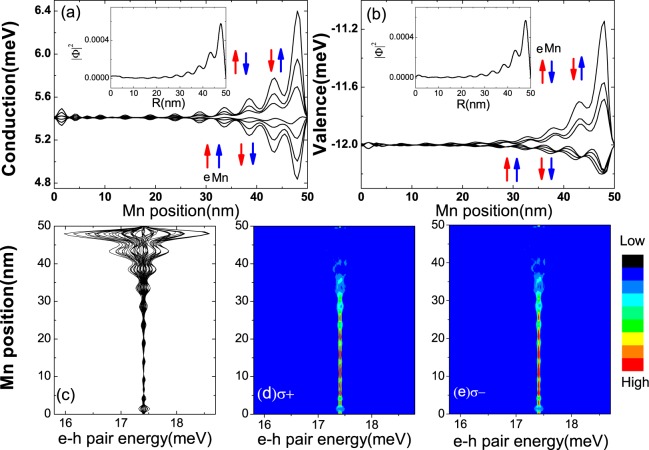
(**a**,**b**) The energies of the edge states from the conduction and valence bands as a function of the position of *Mn*^2+^ ion; the insets in (**a**,**b**) denote the density distributions of edge states in conduction and valence band, respectively. The red (blue) arrows indicate the spin of electrons (*Mn*^2+^ ion). (**c**) The energies of the lowest several levels of the *e* − *h* pairs in the TIQD as a function of the position of *Mn*^2+^ ion, the insets show the electron distributions with *Mn*^2+^ ions in the middle or near the edge of the TIQD respectively. (**d**,**e**) The photoluminescence spectra of the lowest *e* − *h* pair as a function of the position of *Mn*^2+^ ion for the right and left polarized lights *σ*±.

In order to illustrate the importance of *sp-d* interactions on the electronic properties of TIQD, We plot the energies of the edge states at the bottom of conduction band (electron) and at the top of valence band (hole) as a function of the position of *Mn*^2+^ ion in Fig. 4(a,b). As expected, we observe that the energies of both conduction band minimum and valence band maximum oscillate slightly as we move the *Mn*^2+^ ion from the center *r* = 0 *nm* towards the edge as far as *r* = 50 *nm*, Since the *sp-d* interaction between the edge states and magnetic ion depends on the overlap between the wavefunctions of electron/hole and *Mn*^2+^ ion. Hence, there is almost no interaction between the edge states and *Mn*^2+^ ion when the magnetic ion located at the center of TIQD. As the *Mn*^2+^ ion approaches the edge, these energies oscillate intensively with increasing magnitude due to the strongly overlapped wavefunctions between the electron/hole and the *Mn*^2+^ ion. Notably, the energy spectrum of edge states near the bottom of conduction band shows opposite dependence against to the edge states near the top of valence band. It arises from that the opposite signs of the *s-d* and *p-d* exchange interactions. The energies of electrons from bottom conduction band tend to decrease with the same spin orientation between electrons and *Mn*^2+^ ion, while the energies of holes from top valence band tend to increase with the opposite spin orientations between holes and *Mn*^2+^ ion, resulting in the reduced energy gap due to the *sp-d* exchange interaction between the electron/hole and *Mn*^2+^ ion (see the arrows in Fig. 4). We also plot the higher energy levels of electrons and holes, larger energy gaps appear due to the enhanced *sp-d* exchange interaction between the electron/hole and *Mn*^2+^. Notice there are several vibrations of energy and spin splitting as *r* varies from 0 to 50 *nm*. It is the result of the wavefunction vibrations of electron and hole edge states (see the distribution of electron and hole in the inset of Fig. 4(a,b)). We show the lowest electron-hole (e − h) pair energies of TIQDs as a function of the *Mn*^2+^ ion position in Fig. 4(c). When the *Mn*^2+^ ion is located away from the edge of the TIQD, the energy spectrum of *e* − *h* pair are degenerated because of weak interaction between the *Mn*^2+^ ion and the edge states. As the *Mn*^2+^ ion moves towards the edge of the TIQD, the strong *sp-d* interaction lifts the spin degeneracy and splits the energy levels. The *e* − *h* pair energies oscillate as the *Mn*^2+^ ion moves (see Fig. 4(c)), arising from the oscillation of the wavefunction of the ground edge state as indicated by Fig. 4(a,b). It also leads to the oscillating behavior of spin splitting.

Based on the *e* − *h* pair energy, the optical transition rates^36^ of the right and left polarized light *σ*± are calculated as shown in Fig. 4(d,e). When the *Mn*^2+^ ion locates in the center of the TIQD, the optical transition rate of *e* − *h* pairs with right and left polarized light(*σ*±) are almost degenerated and bright. We note that the bright-to-dark transitions occur when the *Mn*^2+^ ion moves from the center (*r* = 0 *nm*) to the edge(*r* = 50 *nm*). The oscillations of the wavefunctions of edge states give rise to the oscillations of *sp-d* interaction strengths and spin splitting energies, that can be manifested by monitoring the bright-to dark transitions as we just discussed. The electrons and holes have opposite *p*–*d* and *s*–*d* interactions, which make the overlap of electron/hole wavefunction from the conduction and valence bands decrease (see the distributions in Fig. 3(e,f)), resulting in the dark transition in spectra. When the *Mn*^2+^ ion moves to the edge of the QD. The conduction and valence bands are strongly coupled by the *sp-d* interaction. There is a bright-to-dark transition in the spectra for both *σ*± polarized lights when the *Mn*^2+^ ion keeps moving to the edge of the TIQD, the wave-function overlap between conduction and valence band decreases (see Fig. 3(e,f)), resulting in the relatively dark in the optical transition spectra. There is no distinguishable strength difference in the optical transition spectra between *σ*± polarized lights. Theoretically, the transition spectra of *σ*± polarized lights depend on the transition matrix and wavefunctions overlaps between carriers in different spin states and the *Mn*^2+^ ion. *Mn*^2+^ doping doesn’t change the transition matrix of *σ*± polarized lights. The wavefunctions overlaps are supposed to be different accounting for the unsymmetrical *sp*–*d* matrix in Four-band Hamiltonian with/without coupling term of the electron/hole-like states respectively (see Eq. 6). Based on our calculation, the difference in optical transition spectra between *σ*± polarized lights is negligible for a single magnetic ion dopant. We could anticipate apparent difference in optical transition spectra between *σ*± polarized lights with more magnetic dopants. The *e* − *h* pair energy and optical transition spectrum behaviors are sensitive to the position of *Mn*^2+^ ion (see Fig. 4(c)). This feature offers a measurement scheme of detecting magnetic dopant via optical transition spectra. Note that the observations here are quite different from that in a conventional semiconductor QD, in which the maximum spin splitting between electrons and the magnetic ion occurs when the the magnetic ion locates in the center of the QD.

**Figure 5:**
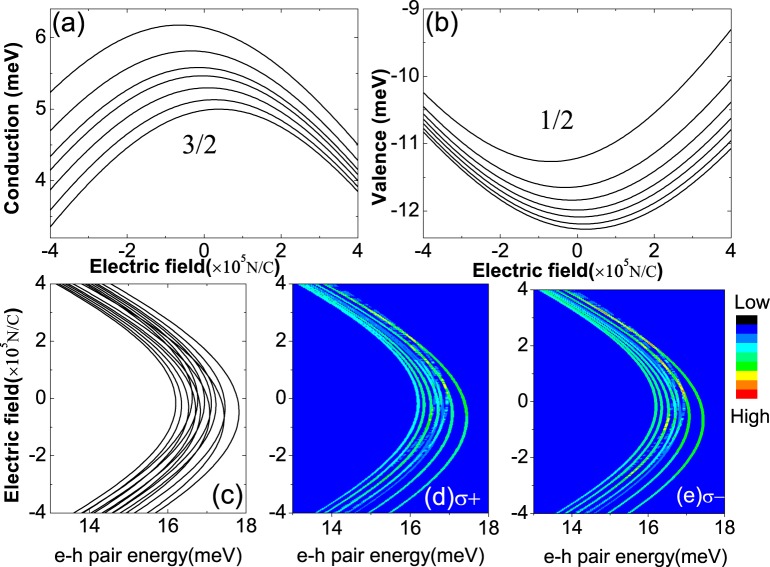
(**a**,**b**) The energies of the edge states from the conduction and valence bands as the function of an in-plane electric field in the TIQD doped with a *Mn*^2+^ magnetic ion at the edge of the TIQD (*R* = 47 *nm*). (**c**) The lowest *e* − *h* pair energies in the TIQD as a function of in-plane electric field. The photoluminescence spectra of the *e* − *h* pair energy as a function of in-plane electric field for right and left polarized lights *σ*± are shown in (**d**,**e**).

The spin splittings of edge states reach maximum when the *Mn*^2+^ ion is moved to the TIQD boundary. By applying an in-plane electric field across the TIQD, the energy spectrum of the edge states near the bottom of the conduction band and near the top of valence band changes significantly as shown in Fig. 5(a,b). When the electric field along the *x* direction increases, the energies of edge states in the bottom conduction band decrease, while that in the top valence band increase duo to the *Stark* effect that has already been observed in Fig. 2. Beside the band gap variations, the spin splittings of the edge states in the conduction band decrease, while that in the valence band increase as the electric field increases. Because the in-plane electric field pulls the electrons of edge states in the conduction band with spin component ±3/2 away from the *Mn*^2+^ ion as shown in Fig. 3(c), resulting in the reduced spin splittings, while it pushes the holes of the edge states in the valence band with spin component ±1/2 towards the *Mn*^2+^ ion as shown in Fig. 3(d), giving rise to larger spin splittings. When the electric field is applied in the opposite direction, we can obtain the opposite modulations of the energy spectrum and spin splittings. So it is an effective way to tune spin splittings of the TIQD edge states by an in-plane electric field. We also plot the energy spectrum of *e* − *h* pair in Fig. 5(c). We can find the same trend of spin splittings as the edge states in conduction band. Note that the maximum spin splittings of lowest *e* − *h* pair states appear when the electric field is small. It comes from the competition between the *p*–*d* splitting of the edge states near the bottom of the conduction band and the *s*–*d* splitting that near the top of the valence band. Since the edge states near the conduction band minimum and valence band maximum are hole-like and electron-like respectively, due to the band inversion. Apparently the hole-like edge states in conduction band play more important roles than the electron-like edge states in valence band with electric field applied along the *x* direction. In the optical transition spectra of Fig. 4(d,e), we can hardly observe the spin splitting when *Mn*^2+^ ion locates near the boundary because it is relatively weak as compared with the case when the *Mn*^2+^ ion is doped in the center of the TIQD. Here we rescale the brightness to view the spin splitting in optical transition spectrum in 5(d,e). We can find the spin splitting varying with the in-plane electric field. The spin splittings can be tunable with applied in-plane electric fields. Here, we propose a novel way to tune spin splitting of edge states in TIQD using the in-plane external electric field as well as a single magnetic dopant. TIQD doped with *Mn*^2+^ ion offer us a promising platform for potential application in a electric control of spin-splitting topological device.

## Conclusions

In summary, we investigate theoretically the energy-angular-momentum dispersions and the spin splittings in the disk-like HgTe TIQDs containing a single *Mn*^2+^ ion. We demonstrate the robustness of edge states with one magnetic dopant. The energy spectra of topological edge states and the corresponding carrier density distributions in such a TIQD can be tuned significantly by the magnetic dopant *Mn*^2+^ ion and a in-plane electric field. Not only the topological edge states in conduction and valence bands show opposite density distributions with in-plane the electric field, but also the *sp-d* exchange interactions between electrons/holes and the magnetic dopant are opposite. We address that the key physics of such manipulations is the coupling between the edge states and the *Mn*^2+^ ion. We then demonstrate that the spin splittings can also be tuned by the in-plane electric field. In optical transition spectra, the spin splittings and bright-to-dark transition can be observed by changing the position of *Mn*^2+^ ion and the strength of the electric field. This electrical controlling capability offers us an efficient way to utilize the TIQD edge states which is robust against the local *Mn*^2+^ ion doping and paves a way to construct the topological photo-electronic device.
